# Automated Calculation of Sequential Organ Failure Assessment (SOFA) Score in the Intensive Care Unit: Algorithm Development, Validation, and Association With 30‐Day Mortality

**DOI:** 10.1111/aas.70205

**Published:** 2026-02-15

**Authors:** Johan Helleberg, Anna Sundelin, Navid Soltani, Ragnar Thobaben, Johan Mårtensson, Olav Rooyackers

**Affiliations:** ^1^ Department of Perioperative Medicine and Intensive Care Karolinska University Hospital Huddinge Stockholm Sweden; ^2^ Section of Anaesthesiology and Intensive Care Medicine, Department of Clinical Sciences Intervention and Technology (CLINTEC), Karolinska Institutet Stockholm Sweden; ^3^ Section of Anaesthesia and Intensive Care, Department of Physiology and Pharmacology Karolinska Institutet Stockholm Sweden; ^4^ Department of Perioperative Medicine and Intensive Care Karolinska University Hospital Solna Stockholm Sweden; ^5^ Division of Information Science and Engineering Digital Futures Faculty, School of Electrical Engineering and Computer Science, Royal Institute of Technology (KTH) Stockholm Sweden

## Abstract

**Background:**

Sequential Organ Failure Assessment (SOFA) score is routinely used in the intensive care unit (ICU) to describe severity of organ dysfunction, for prognostication and sepsis diagnosis, and in clinical trials. Inter‐rater variability and scalability are known challenges in manual assessment. We aimed to develop and validate an algorithm for automatic SOFA calculation and evaluated its predictive abilities on 30‐day mortality.

**Methods:**

Retrospective multi‐center cohort study on all adult patients admitted to four ICUs at the Karolinska University Hospitals in 2015–2018. Data from 2018 collected in one ICU was used for algorithm development. The algorithm was validated by comparing the results of automated SOFA score calculation to those obtained by manual SOFA scoring by experienced intensivists on 300 randomly chosen ICU days from the remaining cohort. Intra‐class correlation coefficient (ICC [95% confidence interval (CI)]) was calculated as primary validation outcome. Area under the receiver operating characteristic curve (AUROC [95% CI]) was used for assessment of 30‐day mortality prediction on the remaining cohort (excluding only the development cohort).

**Results:**

A total of 6953 ICU admissions were included. The algorithm was developed on 613 admissions during 2018. Data from the remaining cohort with 6340 admissions (5076 patients, 36,625 ICU days) were used for mortality prediction. On algorithm validation of the full SOFA score, the ICC was 0.99 (0.98–1.00). For 30‐day mortality, the best predictive abilities were found with maximum SOFA score (AUROC 0.79 [0.78–0.81]) and with SOFA score on Day 2 (AUROC 0.79 [0.77–0.80]).

**Conclusion:**

A trustworthy automated SOFA score dataset can be produced with comprehensive high‐frequency electronic health records curation and rigorous artifact control, with accuracy comparable to manual scoring by senior intensivists. Association between SOFA score and 30‐day mortality in a large, real‐world clinical cohort aligns with findings from previous clinical trials. The results support the use of automated SOFA scoring as a reliable tool for clinical research, quality monitoring, and potentially real‐time clinical decision support.

**Editorial Comment:**

In this article, the authors report results of a retrospective multicenter study, where they used a large cohort of adult ICU patients for developing and validating an automated algorithm for calculating SOFA scores. In validation, they found that the automatically calculated score was comparable to scores manually calculated by experienced clinicians. The automatically calculated maximum SOFA and Day 2 SOFA scores performed well in predicting 30‐day mortality.

## 1 Introduction

The Sequential Organ Failure Assessment (SOFA) score [1, 2] is widely adopted to quantify organ dysfunction and its change over time in ICU patients and supports illness‐severity assessment, prognostication [3], and the sepsis criteria [4]. Beyond clinical practice, the SOFA score is used in clinical trials to define study populations and as an outcome measure.

Previous studies have shown substantial inter‐rater variability in manual SOFA scoring, particularly in the neurologic, cardiovascular, and respiratory subscores [5, 6, 7, 8], and more pronounced when performed by less experienced clinicians [7]. The expansion of electronic health records (EHRs) has enabled the development and deployment of automated algorithms for SOFA calculation [9, 10, 11, 12], which may improve consistency and support longitudinal monitoring [13]. However, few studies have examined the associations between automatically calculated SOFA and mortality, and most have had small sample sizes [8, 11].

A key challenge in automated SOFA calculation from EHR‐derived data is data quality. Erroneous entries, unit inconsistencies, and physiologically implausible values can propagate into calculated scores and compromise reliability. Because critically ill patients exhibit extreme physiologic ranges, simple fixed‐threshold filters may be unreliable and manual data quality checks are increasingly infeasible in high‐frequency ICU data. Data‐driven approaches, including machine learning for anomaly and misclassification detection, offer a means to strengthen preprocessing and may improve the reliability of automated SOFA [13].

We aimed to develop and validate an algorithm for automated SOFA calculation following published recommendations by Lambden et al. [14], with specific attention to reliability in the neurologic, cardiovascular, and respiratory components. We also evaluated the association between automated SOFA and changes in SOFA (delta‐SOFA) during the first 7 days of ICU care and 30‐day mortality. Given that automatic calculation of SOFA score may offer improved consistency and reproducibility, we hypothesized that there may be intrinsic differences in how well SOFA relates to mortality at different timepoints during the ICU stay and sought to determine the best ICU day and cutoff for mortality prediction.

## 2 Methods

### 2.1 Ethical Approval

Swedish Ethical Review Authority (approval number 2019–06203, amendments 2022‐04189‐02 and 2024‐01320‐02) with a waiver of informed consent.

### 2.2 Study Setting

The study was a retrospective cohort study. All adult (age ≥ 18 years) patients admitted to any of the four ICUs at the two Karolinska University Hospitals, Stockholm, Sweden, between January 1, 2015 and December 31, 2018, were included. Admissions transferred from ICUs in other hospitals were excluded to ensure correct ICU‐day. In cases of intra‐hospital transfers between ICUs, consecutive ICU stays were treated as a single episode of intensive care. Admissions to one ICU during 2018 were prespecified as the development set used solely for algorithm development and unit testing of the automated SOFA algorithm; these admissions were excluded from all subsequent validation and outcome analyses to prevent data leakage and ensure unbiased, externally valid estimates. The remaining admissions constituted the study cohort.

### 2.3 Data Acquisition

Data required for SOFA calculation and outcome assessment were extracted from the patient data management system (PDMS; Centricity Critical Care, GE Healthcare, Chicago, IL, USA) and a separate EHR (TakeCare, CompuGroup Medical CGM, Koblenz, Germany). If multiple methods were available for the same variable (e.g., point‐of‐care vs. clinical chemistry laboratory), the worst value was used unless otherwise specified.

### 2.4 Algorithm Development

The SOFA algorithm is described in detail in Appendix S1 and adheres to recent recommendations by Lambden et al. [14]. While the algorithm was developed prior to the publication of SOFA‐2 [15, 16], it was written with the expectation that SOFA‐2 would be updated to include many of the suggestions for SOFA‐implementation from Lambden et al., and to be adaptable to possible changes between SOFA‐1 and SOFA‐2.

Unless otherwise specified, the worst recorded value in each 24‐h window (00:00–23:59) was used for scoring. The renal, coagulation, and hepatic subscores were scored per standard SOFA criteria. As an exception, clinical chemistry sample quality flags were handled in a prespecified manner (Table S1).

For the neurologic component, values of Glasgow Coma Scale (GCS) recorded during ongoing sedation were treated as not assessable for neurologic subscoring, and the last known GCS prior to initiation of invasive mechanical ventilation and sedation was carried forward during the period of ventilation and sedation [14]. Sedation was defined by continuous infusions of prespecified sedatives and mechanical ventilation periods were identified based on ventilator parameters [14]. GCS values obtained ≥ 24 h after cessation of continuous sedation were considered valid for scoring [14]. Within each 24‐h window, if both a carried forward GCS and a valid registered GCS were available, the registered GCS took precedence.

Mislabeling of blood gas sample type is a known source of error in the respiratory SOFA component [10, 17]. We therefore implemented a supervised machine learning model (XGBoost [18]) that was trained in a previous study to remove venous samples mislabeled as arterial prior to scoring [13]. When no arterial blood gas was available, P_a_O_2_ was imputed from saturation using Ellis' equation [19, 20], which has been shown to be accurate in multiple studies, including prospective multicenter ICU studies [14, 20, 21, 22, 23, 24, 25]. For F_i_O_2_ assessment, we used a defined table [14, 26] to estimate F_i_O_2_ for nasal prongs, face mask and similar devices (Table S2).

To mitigate implausible invasive arterial blood pressure readings (i.e., flush artifacts, overdamping, zeroing errors) we implemented a version of a semi‐supervised outlier‐detection framework (XGBOD [27]) to remove such artifacts, (see Appendix S1 for implementation details).

### 2.5 Manual Validation of the Automated SOFA

We performed a retrospective chart review to create a single human reference dataset at the patient‐day level, in accordance with the scoring suggestions by Lambden et al. [14]. From all ICU patient‐days, we randomly sampled 300 patient‐days (allowing multiple days per patient). Two specialist intensivists independently scored all SOFA subscores (blinded to each other and the algorithm) after calibrating on 10 pilot cases. If the two reviewers assigned the same subscore, that value was entered into the reference dataset. If any subscore differed, a third specialist intensivist (blinded to both initial scores) scored only the discrepant items, and this score was used in the reference dataset. The resulting single set of final human subscores was the reference against which the algorithm output was compared. For details on the manual subscores, see Appendix S1.

### 2.6 Statistical Analysis

#### 2.6.1 Missing Data Handling

For the validation, there was no imputation of missing values. For analysis of mortality, we allowed a 24‐h last‐observation‐carried‐forward per component, as suggested in Lambden et al. [14], otherwise assumed normal.

#### 2.6.2 Validation of the Algorithm's SOFA Scores

The primary concordance metric was the two‐way mixed‐effect intra‐class correlation coefficient [28] (ICC) for the total SOFA score. Secondary metrics were the quadratic weighted Cohen's *κ* [29] for the individual SOFA subscores and Bland–Altman analysis with mean difference (bias) and 95% limits of agreement reported [30]. All confidence intervals in the validation process were obtained using a patient‐level clustered bootstrap (1000 resamples, percentile method) to account for possible repeated measurements. No unit‐specific analyses were performed.

#### 2.6.3 Definitions of Derived Measurements

Maximum SOFA (SOFAmax) was defined as each patient's highest SOFA throughout the ICU stay. Delta SOFA (ΔSOFA) at any given day was defined as the difference in SOFA score between that day and ICU Day 1.

#### 2.6.4 Clinical Outcomes and Prognostic Models

The primary outcome to assess prognostic performance was 30‐day all‐cause mortality from the date of ICU admission, with death date extracted from the Swedish population registry. Mortality analyses included only patients with known outcomes and first ICU admissions. To ensure ICU Day 1 reflected the start of the study ICU stay, we excluded admissions transferred into a study ICU from non‐study ICUs.

#### 2.6.5 Sample Size and Power Calculations

We used a two‐stage approach, reflecting the study's two aims (algorithm validation and assessment of predictive performance).

First, we calculated the minimum cohort size needed to detect an absolute difference in area under the receiver operating characteristic curve (AUROC) of 0.05 for 30‐day mortality when comparing SOFA‐measurements (daily SOFA, delta‐SOFA, and maximum SOFA) under the assumption that SOFA‐max would be the best performing metric with an AUROC of 0.80 [3, 31], a two‐sided alpha of 0.05, a 30‐day mortality of 20%, 80% power, and 25% dropout due to missing values; a minimum sample size of 4345 would be required. We selected a calendar period that would yield at least that amount of eligible ICU admissions.

The validation sample size was guided by previous SOFA algorithm validation studies [10, 11] and formally checked for power. We verified at least 80% power to detect an improvement in ICC from an assumed level of 0.85 from manually scored SOFA [5, 32] to ≥ 0.90 (excellent reliability) [28] and a 0.20 increase in the quadratic weighted Cohen's *κ* for the circulatory, neurological, and respiratory scores compared to manual scoring [5, 6], corresponding to one step according to Landis and Koch [33].

#### 2.6.6 Determining the Best Day for Mortality Prediction

Univariate logistic models were fit on each ICU day with SOFA transformed with a natural cubic spline as predictor and 30‐day mortality as outcome. On each day, we built models on the patients present in the ICU at that day only and compared the performance of daily SOFA to maximum and delta‐SOFA. Delta‐SOFA is compared with ICU Day 1. For determining the best performing ICU day, we ran bootstrap resampling with 1000 repetitions and ranked the models by AUROC on each ICU day, and computed the average rank for each ICU day. We then compared the best performing ICU day with the a priori assumed best univariate predictor (SOFAmax) using DeLong's method [34].

Calibration metrics were estimated using optimism‐corrected Harrell's bootstrap, where 1000 models were fit on bootstrap samples on each ICU day, and the model calibration was assessed on the full dataset on that day. Calibration in the large with slope and intercept, as well as Brier's skill score was computed on each day to determine the fractional improvement in prediction of a particular model, compared to a null model that assigns the same probability for all patients.

#### 2.6.7 Determining Best Cutoff and Assessing Non‐Linearity of SOFA

We determined the best cutoffs for SOFA and delta‐SOFA using Youden's criterion [35]. Non‐linearity of the SOFA‐mortality association was tested by comparing a linear model to a natural cubic spline model using a likelihood‐ratio Chi‐square (*χ* ^2^) test.

Continuous data is presented as mean (+/− SD) or median (interquartile range) and was compared between groups with *T*‐test or Wilcoxon Rank‐sum test, as appropriate. Categorical data is presented as count (percentage) and was compared between groups with χ^2^ test. A *p* value < 0.05 was considered statistically significant, and *p* values were Bonferroni‐adjusted to account for repeated testing where applicable.

All statistical analyses were performed using *R* [36], and the SOFA algorithm is written in R, SQL, and C++ [37] using the boost and eigen libraries.

## 3 Results

A total of 6953 ICU admissions met inclusion criteria during the study period. Of these, 613 admissions were used for the construction and refinement of the SOFA algorithm, leaving 6340 admissions (5590 patients, 36,625 ICU days) eligible for algorithm validation and outcome prediction (Table 1). For validation, 300 ICU days (from 275 unique patients) were randomly selected.

**TABLE 1:** Patient characteristics at admission, interventions during ICU stay, and outcomes.

Patient characteristics at admission					
		Overall	Survivors	Non‐survivors	*p*
Age (years)		62 (47–72)	60 (44–70)	71 (60–78)	*p* < 0.001
Sex (male/female)	Female	2173 (38.9%)	1778 (39.2%)	395 (37.7%)	
	Male	3417 (61.1%)	2763 (60.9%)	654 (62.4%)	*p* = 0.375
SAPS 3 points		58.2 (+/− 17.1)	54.0 (+/− 14.8)	76.1 (+/− 14.8)	*p* < 0.001
SOFA score (admission)		5 (3–7)	4 (2–6)	7 (5–9)	*p* < 0.001
Surgery prior to admission		1965 (35.2%)	1735 (38.2%)	230 (21.9%)	*p* < 0.001

Interventions during ICU stay					
Invasive mechanical ventilation (IMV)		3328 (59.5%)	2527 (55.7%)	801 (76.4%)	*p* < 0.001
Vasoactive therapy		3274 (58.6%)	2484 (54.7%)	790 (75.3%)	*p* < 0.001
Continuous renal replacement therapy (CRRT)		578 (10.3%)	386 (8.5%)	192 (18.3%)	*p* < 0.001

Patient outcomes					
Death at 30 days		1049 (18.8%)	0 (0%)	1049 (100%)	*p* < 0.001
Death in the ICU	Yes	604 (10.8%)	10 (0.22%)	594 (56.6%)	*p* < 0.001
Length of stay (LOS) (days)		1.9 (0.8–5.2)	1.88 (0.83–5.3)	2.18 (0.88–4.68)	*p* = 0.652

*Note:* Values are expressed as mean (+/− SD), median (IQR) or *n* (%).

Abbreviations: CRRT, continuous renal replacement therapy; ICU, intensive care unit; IMV, invasive mechanical ventilation; LOS, length of stay; SAPS, Simplified Acute Physiology Score; SOFA, Sequential Organ Failure Assessment.

For the analysis of mortality, 574 patient transfers from other ICUs and 8 admissions without recorded outcome were removed, and only the first admission per patient during the study period was analyzed, leaving 5076 patients (26,813 ICU days) in the cohort for the assessment of the association between SOFA and mortality.

### 3.1 Validation

**FIGURE 1:**
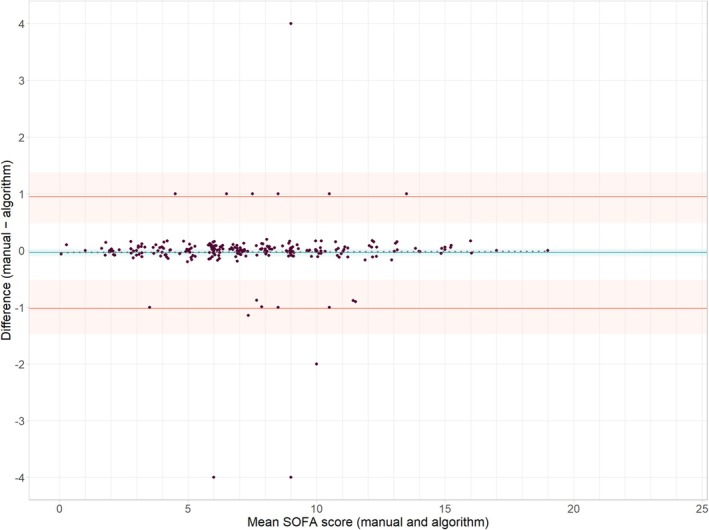
Bland–Altman plot showing bias and limits of agreement. Points are slightly offset at random from actual means and differences to minimize overlap of points and facilitate visualization. Shaded bands represent 95% confidence intervals for bias and limits of agreement.

When comparing the final manual SOFA scores and the algorithm's SOFA scores, the ICC (95% CI) was 0.99 (0.98–1.00), and bias and limits of agreements were −0.03 (−0.09–0.03) and −1.00 (−1.44–−0.56) to 0.94 (0.52–1.35), respectively (Figure 1). The ICC between the two manual raters was 0.98 (0.97–0.99). For the SOFA‐components, the Weighted Cohen's *κ* ranged from 0.95 (95% CI 0.91–0.99) for the neurologic component to 1.00 (1.00–1.00) for the coagulation and renal components (Table 2). Due to missing values, the number of scored days range from 83.3% for the full SOFA score, to 99.0% for the cardiovascular subscore.

**TABLE 2:** Validation results for the individual SOFA components.

Component	Number of scored days (%)	Estimate (95% CI)
Respiratory	283 (94.3%)	0.98 (0.95–1.00)^a^
Coagulation	286 (95.3%)	1.00 (1.00–1.00)^a^
Hepatic	274 (91.3%)	1.00 (0.99–1.00)^a^
Cardiovascular	297 (99.0%)	0.99 (0.98–1.00)^a^
Neurologic	287 (95.7%)	0.95 (0.91–0.99)^a^
Renal	290 (96.7%)	1.00 (1.00–1.00)^a^
Full SOFA	250 (83.3%)	0.99 (0.98–1.00)^b^

*Note:* Validation metrics are weighted Cohen's Kappa (a) and intra‐class correlation (b).

### 3.2 Mortality Prediction

**FIGURE 2:**
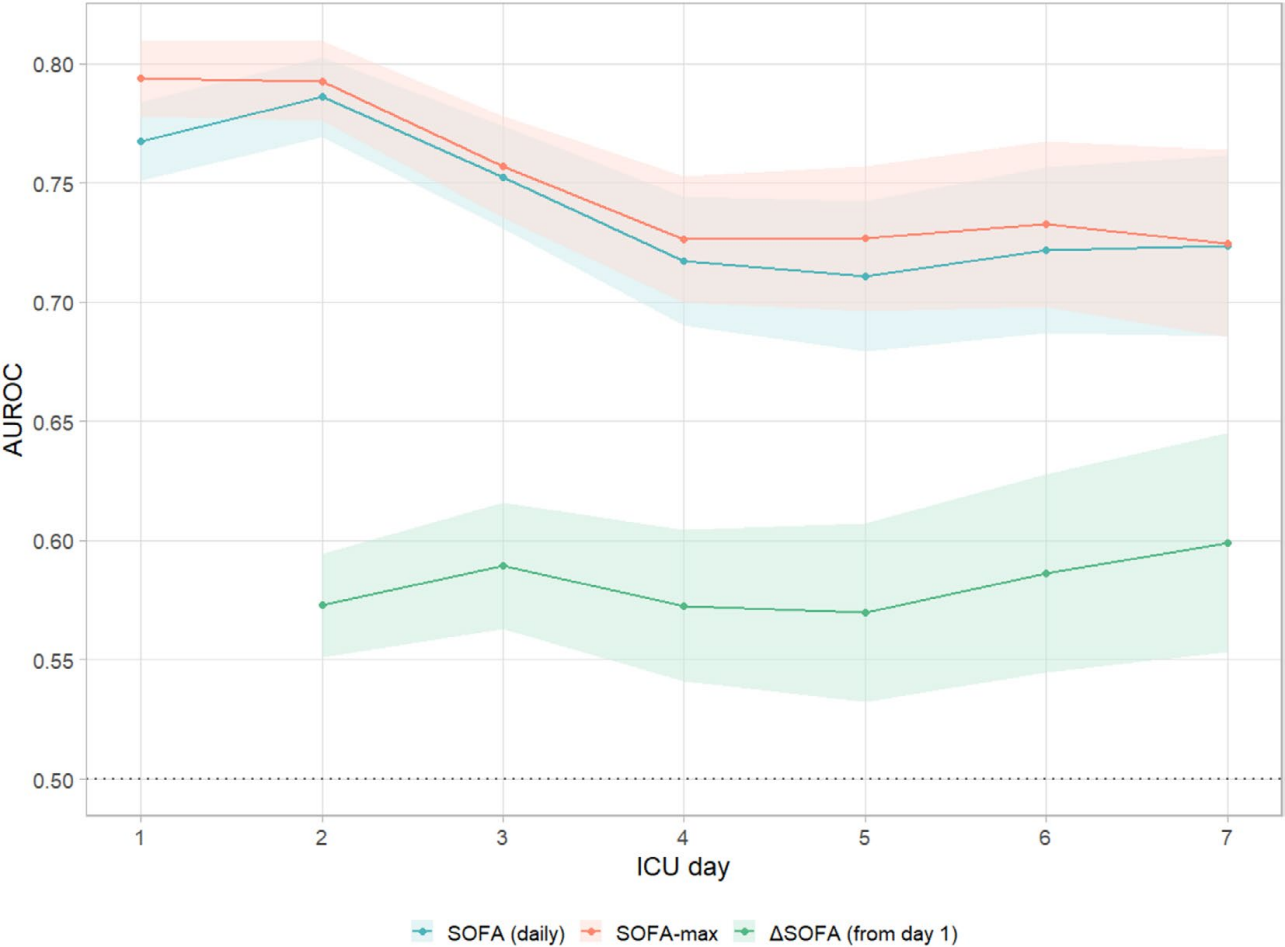
Discrimination for 30‐day mortality per ICU‐day for SOFA, SOFA Maximum (restricted to patients still in the ICU on that day), and ΔSOFA (from ICU Day 1). Shaded bands represent 95% CI for AUROC.

On all ICU days, the total SOFA score discriminated 30‐day mortality better than ΔSOFA (Figure 2). The strongest overall predictor was the maximum SOFA during the ICU stay (SOFAmax), AUROC 0.79 (95% CI, 0.78–0.81). The highest AUROC among single‐day scores occurred on ICU Day 2: 0.79 (0.77–0.80), stable across bootstrap resamples (Table S3). AUROC did not differ between SOFAmax and Day‐2 SOFA (*p* = 0.592).

When comparing daily SOFA with maximum SOFA throughout ICU stay, in the subset of patients present in the ICU on that day, there was no significant difference between maximum SOFA and daily SOFA except on Day 1 (Figure 2). SOFA was a significantly better predictor than ΔSOFA at all time points. The best cutoff for mortality prediction on Day 2 was SOFA ≥ 8, with a sensitivity of 77.3% and a specificity of 67.2%. The best cutoff for ΔSOFA on Day 2 was ΔSOFA ≥ 2, with a sensitivity of 34.4% and a specificity of 78.3% (Table S5).

Calibration assessed with Harrell's optimism‐corrected bootstrap showed intercepts near 0 and slopes with 95% CIs overlapping 1.0 on all ICU days, indicating no evidence of miscalibration or over/underfitting (Table S6). Brier (skill) metrics were highest on ICU Day 2 and decreased thereafter, consistent with the AUROC pattern (Tables 3 and S4).

**TABLE 3:** Discrimination and calibration metrics for SOFA as univariate predictor of 30‐day mortality, per ICU day.

ICU day	Number of patients (*n*)	Prevalence of outcome (%)	AUROC (95% CI)	Brier S score
1	4915	18.5	0.77 (0.75–0.78)	0.155 (0.117–0.191)
2	4495	18.2	0.79 (0.77–0.80)	0.171 (0.132–0.213)
3	2875	20.2	0.75 (0.73–0.77)	0.134 (0.082–0.184)
4	2061	20.2	0.72 (0.69–0.74)	0.097 (0.040–0.154)
5	1597	18.8	0.71 (0.68–0.74)	0.088 (0.011–0.157)
6	1295	17.7	0.72 (0.69–0.75)	0.101 (0.018–0.182)
7	1077	16.8	0.72 (0.69–0.76)	0.097 (0.000–0.184)

A likelihood‐ratio *χ* ^2^ test comparing spline versus linear terms for SOFA was significant on ICU Days 2 and 7 after Bonferroni correction, supporting a non‐linear association between SOFA and log‐odds of mortality (Figure S1). On Day 2, the local odds ratio for a one‐point SOFA increase peaked at SOFA = 6, whereas the largest absolute increase in predicted mortality per one‐point increment occurred at SOFA = 10 (Figures S1–S4).

## 4 Discussion

### 4.1 Key Findings

We developed and validated an EHR‐derived SOFA algorithm that achieved excellent agreement with expert manual scoring (ICC 0.99; no meaningful bias; narrow limits of agreement). This agreement is due to extensive efforts to ensure high quality input data. For prognosis, SOFAmax had the highest overall discrimination for 30‐day mortality, but SOFA on ICU Day 2 performed equivalently (AUROC 0.786 vs. 0.79, *p* = 0.592), providing an early substitute for SOFAmax, useful for risk‐stratification or enrollment in trials. Across all time‐points, daily SOFA outperformed ΔSOFA, while ΔSOFA ≥ 2 remained the optimal marker for deterioration. The SOFA‐mortality association was day‐dependent and non‐linear, with the impact of a one‐point SOFA increase varying across the SOFA range, reinforcing the need to report ICU day and to model non‐linear effects when interpreting SOFA.

### 4.2 Relationship With Previous Studies

Our findings are consistent with previous literature on SOFA score validity and prognostic performance. Several studies have reported agreement between electronically calculated and manually scored SOFA values, with our results demonstrating higher concordance [9, 11, 17]. While the SOFA score was originally developed to describe the extent of organ dysfunction rather than to predict mortality, its association with clinical outcomes has been repeatedly demonstrated [1, 3]. The result showing ΔSOFA ≥ 2 as a deterioration marker is also described in the work leading to the criteria for Sepsis‐3‐diagnosis [4, 38]. In line with earlier findings, we observed that maximum SOFA was the strongest predictor of 30‐day mortality, and that Day 2 SOFA had the highest predictive value among individual days during the first ICU week [3]. These patterns have been reported in both manually and electronically derived SOFA scores and lend further support to the external validity of our results, as well as the accuracy of the data processing pipeline used.

Compared to previous studies, our algorithm incorporates more extensive handling of outliers and missing or faulty data. The high level of agreement observed in this study may in part reflect the effects of careful data preprocessing and iterative refinement of the algorithm. While machine learning has been used in related contexts, no prior study has applied it as extensively for automatic SOFA scoring. In addition, no prior study on automatic SOFA score has reported adherence to recent suggestions for standardized SOFA calculation [14], particularly in how the GCS values are handled during sedation and mechanical ventilation.

### 4.3 Strengths and Limitations

This study has several strengths. The cohort is large, includes data from two centers including two general ICUs, one neuro ICU, and one thoracic ICU, and has a low rate of exclusions, supporting generalizability. Data was recorded at high temporal resolution and is of high quality, enabling detailed SOFA scoring. Manual validation was rigorously performed by two to three intensive care specialists per case, strengthening the reliability of the reference standard. The algorithm itself was developed through extensive iteration and incorporates advanced machine learning techniques applied in novel ways to address challenges such as mislabeled or missing data. Furthermore, we have demonstrated the relationship between the scores and patient outcomes, supporting its utility to the bedside clinician.

This study also has limitations. Patients with longer ICU stay are overrepresented in the validated ICU days, which may limit generalizability to short‐stay or less severely ill populations. SOFA scoring was performed retrospectively, and although based on complete clinical data, true bedside assessments cannot be fully replicated. Real‐time scoring by clinicians would be ideal but is rarely feasible in practice, as it would require continuous presence and may introduce observer effects or clinical intervention bias. Using GCS assessed 24 h after discontinuation of sedation may still not reflect true neurological function, as residual sedative effects can persist beyond 24 h. Moreover, assessment of the verbal component of the GCS in intubated patients is prone to bias. Additionally, while the algorithm was evaluated in a multi‐center setting, it remains to be tested in other healthcare systems or with different EHR infrastructures to assess broader applicability.

### 4.4 Clinical Implications and Future Research

As high‐resolution EHR data is becoming increasingly important in clinical research and decision‐making [17, 39] there is increased value in reliable SOFA automation. Our results suggest that accuracy comparable to practicing clinicians is possible and support the use of automatic scored SOFA on Day 2 as a practical replacement for maximum SOFA when future information is unavailable (e.g., for early risk stratification and mitigation or screening for clinical trials).

SOFA scoring remains central to ICU research and has gained further prominence since its inclusion in the Sepsis‐3 definition in 2016 [4]. Our algorithm, developed in close collaboration with practicing intensivists, has already gained interest from ICU researchers at our participating centers. By making the algorithms openly available, we aim to facilitate its implementation in other cohorts and clinical environments. Given its accuracy and attention to detail, we believe it may be useful not only in intensive care settings but also in high‐acuity hospital wards and potentially in future outpatient or remote monitoring contexts. Our algorithm will potentially be adaptable to the SOFA‐2 criteria [15, 16]. As high‐resolution physiological data becomes more widely available—through hospital systems or wearable devices—we anticipate a growing role for automated scoring tools such as ours in supporting timely and accurate clinical assessments.

## 5 Conclusion

SOFA can be calculated automatically from EHR data with accuracy comparable to manual scoring by senior intensivists. Additionally, we show that the relationship between SOFA and mortality is non‐linear and varies across time, with the best predictive performance found at ICU Day 2. These results support the use of automated SOFA scoring as a reliable tool for clinical research, quality monitoring, and potentially real‐time clinical decision support.

## Author Contributions

J.H. was the principal designer of the study, wrote all code, performed all statistical analyses, performed literature review, and wrote the final manuscript together with A.S. A.S. performed all the manual classifications of SOFA scores, suggested the scientific study of the blood gas classification problem, performed literature review, and wrote the final manuscript with J.H. N.S. manually classified the SOFA scores and aided in writing the final manuscript. R.T. reviewed all code and suggested improvements and aided in writing the final manuscript. J.M. co‐funded the study, supervised A.S., contributed to the feature engineering, contributed to the interpretation of the results, and aided in writing the final manuscript. O.R. co‐funded the study, supervised J.H., contributed to the design of the study, contributed to the interpretation of the results, and aided in writing the final manuscript.

## Funding

The authors have nothing to report.

## Disclosure

The authors confirm that the work described has not been published before, that it is not under consideration for publication elsewhere, and that its publication has been approved by the responsible authorities at the institutions where the work is carried out.

## Ethics Statement

The study was approved by the Swedish Ethical Review Authority (approval number 2019‐06203) with a waiver of informed consent. The study was submitted to, and approved by, a national ethics committee. The statements from the Swedish Ethical Review Authority (in Swedish) are attached as related files.

## Consent

Individuals cannot be identified and consent for publication is not deemed required. The interests of the authors have not been over‐prioritized. The best interests of the individual have been considered to the full extent possible. The authors warrant that their contribution is original and that they have full power to make this consent.

## Conflicts of Interest

The authors declare no conflicts of interest.

## Supporting information


**Appendix S1:** aas70205‐sup‐0001‐AppendixS1.docx.

## Data Availability

The data that support the findings of this study are not openly available due to reasons of sensitivity and are available from the corresponding author upon reasonable request. Data are located in controlled access data storage at Karolinska Institutet. Example from: https://doi.org/10.1186/s12910‐022‐00758‐z.
