# Supplementary material for: Automated Calculation of Sequential Organ Failure Assessment (SOFA) Score in the Intensive Care Unit: Algorithm Development, Validation, and Association With 30‐Day Mortality
Source: Acta Anaesthesiol Scand. 2026 Feb 15;70(3):e70205. doi: 10.1111/aas.70205 (PMC12907534; doi:10.1111/aas.70205)
Supplement: Supplementary file 1 — Appendix S1: aas70205‐sup‐0001‐AppendixS1.docx. [file AAS-70-0-s001.docx]

Supplementary Appendix

# Automated Calculation of Sequential Organ Failure Assessment (SOFA) Score in the Intensive Care Unit : Algorithm Development, Validation, and Association with 30-day Mortality

## Methods

### Manual reference standard

#### Design and blinding

Each calendar day (index day, 00:00–23:59) was independently reviewed by two senior intensivists who were blinded to each other, to the automated algorithm, and to outcomes. Disagreements were resolved by a third senior intensivist. Inter-rater agreement was calculated for each organ and for total SOFA. Index days were randomly sampled from all ICU days.

#### Data and selection rule

Reviewers used the full EHR, including flowsheets, laboratory results, device logs, and medication administration records. For each organ, they selected the worst credible underlying values after cross-checking sources and context. Non-credible values were prespecified according to Nielsen et al^1^. Obvious artifacts were excluded through clinical expertise. Reviewers did not assign subscores. The study algorithm mapped the selected values to SOFA categories using fixed, published thresholds.

### Organ-specific ascertainment

#### Respiratory

- PaO₂/FiO₂ used FiO₂ aligned to ABG timing. Reviewers considered both with and without mechanical ventilation and retained the worse applicable set.
- FiO₂ for HFNC, CPAP, or NIV came from nurse-entered device settings.
- Estimated F_i_O_2_ in patients receiving ventilatory support using facemasks is shown in Table S2.
- If no ABG was available, SpO₂/FiO₂ substitution followed the thresholds from Pandharipande et al^2^.
- Samples labeled arterial but inconsistent with clinical context were excluded as likely venous after expert review.
- PaO₂ was considered related to mechanical ventilation if the blood gas analysis time coincided with documented mechanical ventilation.

#### Coagulation (platelets)

- The lowest credible platelet count within the index day, informed by trend review across the ICU stay.

#### Hepatic (bilirubin)

- The highest credible bilirubin within the index day, with trend and context checks for plausibility.

#### Cardiovascular

- Catecholamines and vasopressors were converted to norepinephrine base equivalents according to Goradia et al^3^ and Lambden et al^4^.
- Actual body weight from the first 24 hours, plausibility-checked, was used for dose normalization.
- Arterial and non-invasive blood pressures were cross-validated for internal coherence.
- Vasoactive agents must be infused for at least one hour to qualify for a particular score tier, as suggested by Vincent et al^5^.

#### Neurologic (Glasgow Coma Scale [GCS])

- With continuous sedation, the last reliable pre-sedation GCS was carried forward through sedation and for 24 hours after cessation. If sedation was paused for more than 24 hours, new GCS values were used, per the recommendations of Lambden et al^4^. Neuromuscular blockade is not used without sedation in these units.
- In mechanically ventilated intubated/tracheostomized patients, we assigned GCS verbal = 1, per local standard practice.

#### Renal (creatinine and urine output)

- Worst credible creatinine on the index day, trend-informed.
- Urine output was linearly scaled to 24 hours when incomplete and was vetted for plausibility, for example recognizing an initial large void after catheter insertion.

#### Real-world workflow

For this study, two specialist physicians independently scored the validation cohort retrospectively using data from both the EHR and the PDMS, with a third intensivist acting as arbiter to create a consensus when applicable.

In routine clinical practice at our ICUs, SOFA scoring is performed once daily by the responsible intensivists for each admitted patient. The scoring is supported by the PDMS/EHR infrastructure, which aggregates both automatically captured data from bedside monitors, ventilators, and infusion pumps, as well as manually entered observations and assessments documented by specialist nurses and assistant nurses (e.g. neurological assessments, urine output, weight and other charted clinical findings). This integrated workflow allows the intensivist to review the most recent 24-hour data and finalize the daily SOFA score as part of standard multidisciplinary ICU rounds. The participating intensivists in the study were well acquainted with performing SOFA scoring as part of routine clinical practice.

### Quality and traceability

Selections were entered in predefined fields, one worksheet per reviewer, with optional comments for rationale. Artifact exclusion relied on cross-source consistency and clinical judgment. The algorithm then applied fixed rules, including the Vincent, Pandharipande and Lambden specifications, to derive organ subscores and the total SOFA.

### Algorithm details and development

A research database was constructed for the included patients based on raw database tables extracted from the PDMS warehouse for the selected patients. The developers had access to the variable descriptions and table fields in the clinical database when determining which variables were needed for the SOFA algorithm.

The SOFA algorithm queries a database with predefined queries that retrieves the information needed for each component. When the same measurement is available from multiple sources, the algorithm is designed to make them comparable (for example, harmonizing units of measurement before aggregation).

#### Coagulation, Hepatic and Renal scores.

Numeric codes corresponding to certain statements from point-of-care blood gas analysis were parsed and the algorithm deletes or keeps values based on these. For clinical chemistry results, values below or above the lower limits of quantification (for example, thrombocyte count <5 * 10^9^/mL) were assumed to be equal to the lower or higher limits for the purpose of SOFA scoring. Additionally, standard comments from the clinical chemistry laboratory were parsed with regular expression matching and treated accordingly.

Table S1. Clinical chemistry sample quality flags were handled in a prespecified manner.

| Lab statement | Source | Action | Notes |
| --- | --- | --- | --- |
| Value greater than reference range | Point-of-care analysis | Keep |  |
| Value below reference range | Point-of-care analysis | Keep |  |
| Value below critical range | Point-of-care analysis | Keep |  |
| Value above critical range | Point-of-care analysis | Keep |  |
| Calibration error | Point-of-care analysis | Delete |  |
| Quality Control not passed | Point-of-care analysis | Delete |  |
| Turbidity higher than 5%, too high for reliable measurements | Point-of-care analysis | Delete |  |
| Sample error | Point-of-care analysis | Delete |  |
| Base Excess outside measurement range | Point-of-care analysis | Keep |  |
| Old sample | Point-of-care analysis | Delete |  |
| Discrepancy between pO2 and sO2-measurements | Point-of-care analysis | Delete |  |
| Sample error | Clinical chemistry | Delete |  |
| Replaces previous result | Clinical chemistry | Detect previous result as referenced |  |
| Sample from another patient | Clinical chemistry | Delete | Found through regular expression matching |
| Sample from wrong patient | Clinical chemistry | Delete | Found through regular expression matching |
| Aggregation of thrombocytes, recommend resampling in citrate | Clinical chemistry | Delete | Found through regular expression matching |

Table S2. Estimated F_i_O_2_ in patients receiving ventilatory support using facemasks. Adapted from the International study of the prevalence and outcomes of infection in intensive care units^7^ and visualized by Lambden et al^4^.

| Estimated F_i_O_2_ in patients supported with oxygen via facemask | | | | |  |
| --- | --- | --- | --- | --- | --- |
| Flow rate (l/min) | 5 | 6-7 | 7-8 |  |  |
| Estimated F_i_O_2_ | 0.4 | 0.5 | 0.6 |  |  |
| Estimated F_i_O_2_ in patients supported with oxygen via facemask with reservoir bag | | | | | |
| Flow rate (l/min) | 6 | 7 | 8 | 9 | 10+ |
| Estimated F_i_O_2_ | 0.6 | 0.7 | 0.8 | 0.9 | 0.95 |

#### Decimal error correction of manually input data

Data entered manually in fields was checked with an algorithm written to detect decimal point errors. For a given variable such as weight or height, any measurement outside a predetermined plausible range was separated from those measurements within the range. Then, the mean and standard deviation of the plausible measurements were computed. Then, for each measurement outside the plausible range, a decimal shift of up to 4 orders of magnitude in either direction was attempted, if any such decimal shift resulted in the transformed value falling within the range of mean +/- 3 SD of the observed values, the transformed value replaced the original value (for example, entering 1.69 cm as height would be transformed to 169 cm, or a pH of 7403 would be interpreted as 7.403).

#### Weights and heights

To determine the weight (for the purpose of dosing of sedative or vasoactive drugs), the EHR data for all weights and heights measured after the age of 18 were retrieved. The decimal input shift logic was applied as described above. Then, the algorithm tries to detect heights erroneously being written in the weight field and switching those, specifically if Height < 120 cm and Weight > 150 kg exist with the same timestamp, and no other heights < 120 exist after the age of 18, switch the fields for height and weight. Then, a rolling Hampel filter (marking values outside the range of median +/- 3 median absolute deviations) was applied to the weights over the first week of ICU stay. The first measured weight not marked by the Hampel filter (i.e. within 3 robust estimated standard deviations of the observed data) was considered the patients admission weight and used in calculations.

#### Central nervous system scoring

Due to the relative sparsity of CNS scores, no specific outlier detection code was implemented to avoid removing possible true values. Instead, partial GCS values were completed with the last known observation of the other components. Furthermore, continuous infusions of sedative drugs known to be used at the ICUs in the study (thiopental, ketamine, esketamine, midazolam, isoflurane, sevoflurane, remifentanil, dexmedetomidine, propofol) were determined from the settings of infusion pumps, and GCS values recorded while at least one sedative drug infusion was ongoing in a dose consistent with the range used for sedation (not analgesia, in the case of esketamine and ketamine) were considered invalid and not used for scoring. Periods of invasive mechanical ventilation (IMV) were determined from ventilator settings, and during overlapping periods of IMV and any sedative infusions as described above, the last known GCS values prior to the initiation of IMV and sedation was carried forward throughout the period. If no GCS was recorded prior to the initiation of IMV and sedation, a GCS value of 15 was assumed and carried forward.

#### Respiratory scoring

As sample type errors have been described (venous samples marked as arterial), it was assumed that this would be a problem in our database too. Given the extreme range of physiological ranges expected in critically ill patients, we expected fixed rule base filters to be inadequate and performed a separate substitute with the aim of using supervised machine learning to correctly determine sample type of blood gas samples from ICU patients. We refer to that study and its supplementary material for details^6^.

For F_i_O_2_ assessment, we used a defined table^4; 7^ to estimate F_i_O_2_ for nasal prongs, face mask and similar devices, (Table S1). From ventilator data, we used the measured rather than set F_i_O_2_ when available. F F_i_O_2_ values were shifted 5 minutes backwards in time in order to reflect the F_i_O_2_ at sample time, rather than at the time of complete analysis in the point of care analyzer.

When no P_a_O_2_-values were available, P_a_O_2_ was estimated from peripheral saturation (S_p_O_2_) using Ellis’s equation^8^.

#### Cardiovascular SOFA

Arterial blood pressure (ABP) is recorded in the database at a frequency of about 1 per 2 minutes (≈720 per 24 hours). Because invasive blood pressure monitoring is prone to artifacts (sampling/zeroing, damping, kinked tubing, patient/transducer motion etc.) and the SOFA cardiovascular subscore uses the single lowest MAP in each 24-hour window, it was assumed that artifact values would be common and easily push patients from subscore 0 to 1 if not detected and removed with extremely high fidelity. Likewise, due to the rapid fluctuations seen in the ICU, including sudden cardiac arrest, we assumed that naïve implementations (rolling means +/- standard deviations and similar methods) would not provide the desired sensitivity and specificity to be able to remove only artifact values with high accuracy. Thus, a semi-supervised anomality detection pipeline based on the XGBOD^9^ framework was implemented to discard spurious lows before selecting the daily minimum for scoring.

After filtering, the lowest mean arterial pressure (MAP) in each time window was used for scoring; if both non-invasive and invasive measurements were available, the lowest MAP regardless of method was used. Vasoactive infusion rates were registered in the PDMS every 15 minutes plus every time the rate was changed. For vasoactive support we followed the recommendations by Lambden et al^4^ in calculating the maximum concurrent norepinephrine base equivalent dose^3; 4^ (NEE in µg/kg/min). It was calculated by summing all ongoing infusions of vasoactive drugs within the 24-hour window according to published equivalence tables. In line with the original SOFA only vasoactive infusions exceeding 60 minutes were used^5; 10^. The cardiovascular subscore was then assigned from MAP and NEE per standard SOFA cut points.

##### XGBOD framework in brief

We refer to the original publication for a thorough analysis of the properties of the algorithm^6; 9^. Let $\text{X}\in\mathbb{R}^{nxp}$ be the original feature matrix for which the known labels $\text{y}\in\left\{ 0,1 \right\}^{n}$ are known (0 = normal measurement, 1 = anomaly). For an unsupervised outlier detection function $\phi\left( \cdot\right)$, define the score vector $s = \phi\left( \boldsymbol{X} \right)\in\mathbb{R}^{n}$. An accuracy function $A\left( s,y \right)$quantifies how well s separates classes. In the original publication, AUROC was suggested as A, but in the present pipeline we used area under the precision-recall curve (AUCPR) due to the expected class imbalance.

Let $\mathcal{S=}$ denote the set of score vectors derived from a number m of outlier algorithm and hyperparameter combinations. A greedy feature selection process is then applied to avoid having to search the full feature space, where the features are ranked by discounted accuracy DA, defined as:

$DA(s^{i}) = \frac{A(s^{i},y)}{\sum_{s^{j}\mathcal{\in S}} \rho\left( s^{i}, s^{j} \right)}$, where ρ represents Pearson’s correlation.

Greedy selection starts from $\mathcal{S=\emptyset}$, adds the feature with highest $A\left( \cdot\right)$, then iteratively adds the feature with highest discounted accuracy with relation to the current $\mathcal{S}$ until some predetermined number of features k < m is reached.

##### Feature space and distance computation

Each ABP was embedded in a 4-dimensional space;

1. t, time (seconds)
2. MAP, mean arterial pressure (mmHg)
3. (Systolic – MAP) / MAP
4. (MAP – Diastolic) / MAP

The unitless 3^rd^ and 4^th^ dimensions were defined to give information about the waveform properties at each time point. Distances were computed in weighted Euclidian space, with weights tuned in an algorithm-specific grid search.

##### Training dataset for algorithm development

A dataset enriched for challenging cases was generated by sampling 6-hours ABP segments centered on observed MAP < 40 or MAP > 180 that were not flagged by Tukey’s fences, in other words that were likely to be either difficult to detect artifacts or truly extreme MAP. The training dataset consisted of 86400 MAP points sampled from the set of patients available for unit testing of the SOFA algorithm (thus without any data overlap with the study cohort). A specialist in intensive care (who did not participate in the validation of the future SOFA scores later on) manually labeled each time point as correct or faulty measurements. These labels formed y.

We implemented following algorithms to generate scores

- Local Outlier Factor (LOF)^12^
- Connectivity-based Outlier Factor (COF)^13^
- Local Outlier Probability (LoOP)^14^
- k-Nearest Neighbors average distance (kNN-avg)^15; 16^
- Local regression residuals (time🡪map), Standardized Residuals of local linear regression in the (t, MAP)-plane, with a grid search of window size and polynomial degree of t

For each method/hyperparameter/weight-combination we computed a score vector $s^{i}$ for inclusion in the XGBOD^9^ pool. Algorithm parameters (such as k for k-NN) were determined to maximize the AUCPR of the outlier algorithm.

##### Model selection and training

Greedy selection was a-priori limited to 10 features (including the original MAP, Systolic and Diastolic), to reduce the size of the matrix for the XGBoost predictions in large data sets. XGBoost was trained with a grid-search of hyperparameter space using 5-fold cross-validation. Finally, a XGboost model was trained on the full dataset (86400 data points) with the best hyperparameters from the cross-validation. The decision cutoff was determined by optimizing F1-score on the PR-curve.

##### Decision rule for SOFA scoring

Each ABP point receives an anomaly probability p̂. Points with p̂ > 0.265 are discarded before choosing the lowest MAP for SOFA scoring.

##### Implementation details

All unsupervised models and the local regression were implemented in C++ with parallel processing to enable high throughput for large datasets. Distance computations in all algorithms share a common weighted Euclidian template function, neighborhood searches are batched to optimize cache locality.

## Results

ICU day 2 is showing the best mortality predictive abilities in our material (Table S3).

Table S3. Rankings in mortality predictive abilities per ICU-day 1 through 7 across the 1000 bootstraps.

| ICU- day | Repeats | P(best) | P(top 3) | Mean rank | AUC (95% CI) |
| --- | --- | --- | --- | --- | --- |
| 1 | 1000 | 0.000 | 0.998 | 2.084 | 0.767 (0.750 - 0.784) |
| 2 | 1000 | 1.000 | 1.000 | 1.000 | 0.786 (0.769 - 0.802) |
| 3 | 1000 | 0.000 | 0.924 | 3.023 | 0.753 (0.732 - 0.774) |
| 4 | 1000 | 0.000 | 0.001 | 5.595 | 0.717 (0.690 - 0.744) |
| 5 | 1000 | 0.000 | 0.000 | 6.248 | 0.711 (0.680 - 0.742) |
| 6 | 1000 | 0.000 | 0.028 | 5.105 | 0.722 (0.686 - 0.754) |
| 7 | 1000 | 0.000 | 0.049 | 4.945 | 0.724 (0.686 - 0.758) |

Non-linearity was determined and assessed each day of the first ICU week (Table S4).

Table S4. Determining and assessing non-linearity of SOFA, per ICU-day 1 through 7.

| ICU-day | df_diff | Likelihood ratio | p, raw | p, Bonferroni |
| --- | --- | --- | --- | --- |
| 1 | 2 | 3.794 | 0.15 | 1 |
| 2 | 2 | 11.973 | 0.003 | 0.018 |
| 3 | 2 | 9.322 | 0.009 | 0.066 |
| 4 | 2 | 5.561 | 0.062 | 0.434 |
| 5 | 2 | 3.483 | 0.175 | 1 |
| 6 | 2 | 5.265 | 0.072 | 0.503 |
| 7 | 2 | 11.959 | 0.003 | 0.018 |

The best cutoff for ΔSOFA on day 2 was ΔSOFA ≥ 2, with a sensitivity of 34.4% and a specificity of 78.3%, (Table S5).

Table S5. Cutoffs in mortality predictive abilities SOFA and delta-SOFA per ICU-day 1 through 7 including specificity and sensitivity analyses.

|  | SOFA |  |  | DeltaSOFA |  |  |
| --- | --- | --- | --- | --- | --- | --- |
| ICU day | Cutoff | Sensitivity | Specificity | Cutoff | Sensitivity | Specificity |
| 1 | 7.5 | 0.724 | 0.68 |  |  |  |
| 2 | 7.5 | 0.773 | 0.672 | 0.5 | 0.433 | 0.671 |
| 3 | 8.5 | 0.671 | 0.714 | 1.5 | 0.344 | 0.783 |
| 4 | 7.5 | 0.698 | 0.619 | 0.5 | 0.482 | 0.643 |
| 5 | 7.5 | 0.69 | 0.622 | 2.5 | 0.259 | 0.857 |
| 6 | 8.5 | 0.594 | 0.718 | 2.5 | 0.277 | 0.853 |
| 7 | 7.5 | 0.696 | 0.612 | 1.5 | 0.356 | 0.781 |

When assessing calibration with optimism-corrected Harrel’s bootstrap^17^ of calibration in the large (CITL), we found no evidence of bias or under- or overfit on any of the ICU days, with intercept close to 0 and the confidence interval of slope overlapping 1.0 at all times (supplementary table S6).

Table S6. Calibration in the large (CITL) per ICU-day 1 through 7.

| ICU day | Number of patients | Prevalence of outcome | CITL - intercept | CITL - slope | Brier Score | Brier Skill score |
| --- | --- | --- | --- | --- | --- | --- |
| 1 | 4915 | 0.185 | -0.002 (-0.075 - 0.072) | 0.998 (0.920 - 1.076) | 0.128 (0.122 - 0.133) | 0.155 (0.117 - 0.191) |
| 2 | 4495 | 0.182 | 0.001 (-0.081 - 0.080) | 0.997 (0.923 - 1.081) | 0.123 (0.117 - 0.129) | 0.171 (0.132 - 0.213) |
| 3 | 2875 | 0.202 | 0.003 (-0.091 - 0.102) | 0.996 (0.889 - 1.111) | 0.139 (0.131 - 0.148) | 0.134 (0.082 - 0.184) |
| 4 | 2061 | 0.202 | 0.000 (-0.113 - 0.115) | 0.992 (0.851 - 1.149) | 0.146 (0.136 - 0.155) | 0.097 (0.040 - 0.154) |
| 5 | 1597 | 0.188 | 0.005 (-0.126 - 0.147) | 0.989 (0.822 - 1.179) | 0.139 (0.129 - 0.151) | 0.088 (0.011 - 0.157) |
| 6 | 1295 | 0.177 | -0.003 (-0.156 - 0.162) | 0.978 (0.799 - 1.181) | 0.131 (0.119 - 0.143) | 0.101 (0.018 - 0.182) |
| 7 | 1077 | 0.168 | 0.001 (-0.171 - 0.168) | 0.977 (0.782 - 1.223) | 0.126 (0.114 - 0.140) | 0.097 (0.000 - 0.184) |

A likelihood-ratio χ² test comparing spline versus linear terms for SOFA was significant on ICU days 2 and 7 after Bonferroni correction, supporting a non-linear association between SOFA and log-odds of mortality (Supplementary Figure S1). On day 2, the local odds ratio for a one-point SOFA increase peaked at SOFA = 6, whereas the largest absolute increase in predicted mortality per one-point increment occurred at SOFA =10 (Supplementary Figures S1, S2, S3 and S4).


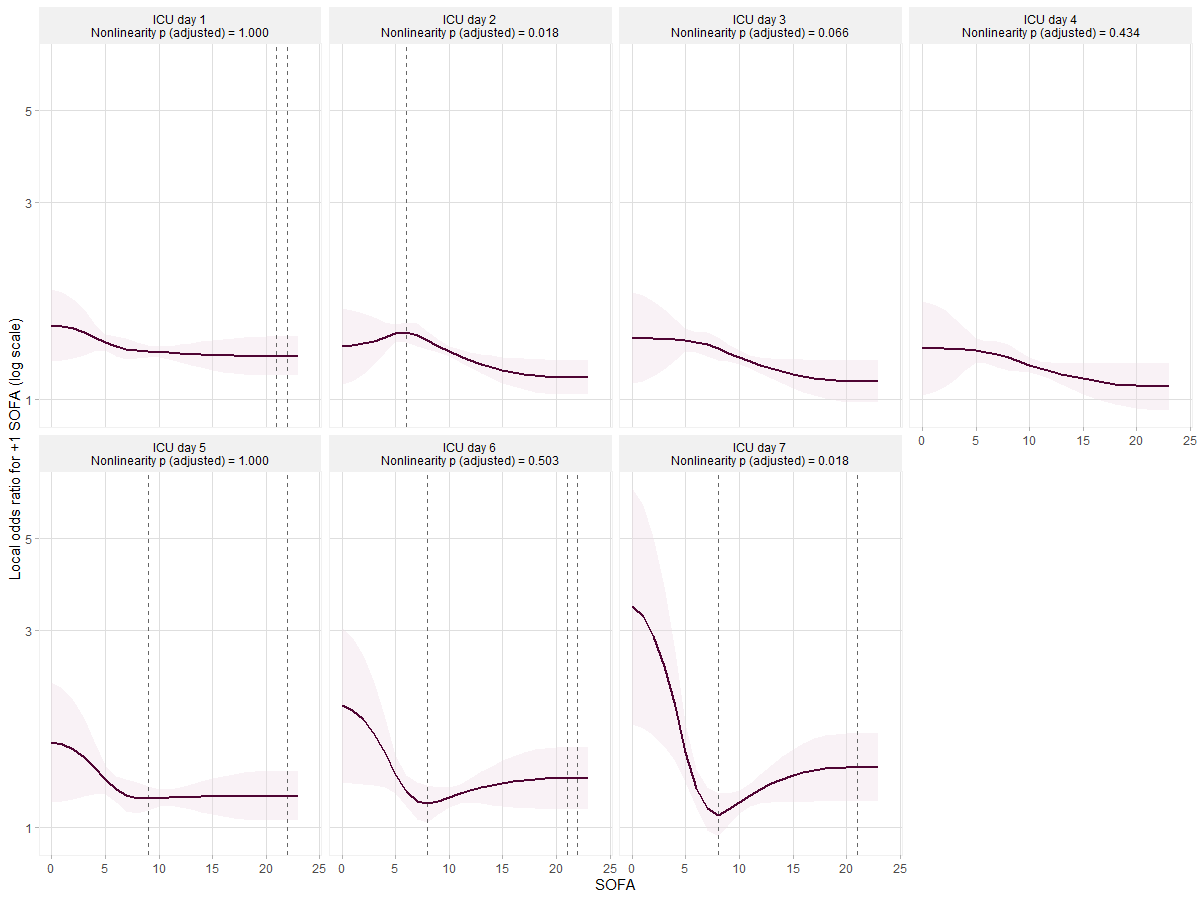


Figure S1. Odds ratio for mortality related to increase of SOFA +1 per ICU-day 1 through 7. In ICU-day 1 an increase of +1 does not generate a major difference in mortality. In day 2, an increase of +1 has the greatest effect on mortality odds ratio when SOFA is about 6 points. The reference interval is larger due to fewer observations in the later stages of intensive care, and closer to the ends of the intervals (very low and very high SOFA scores).


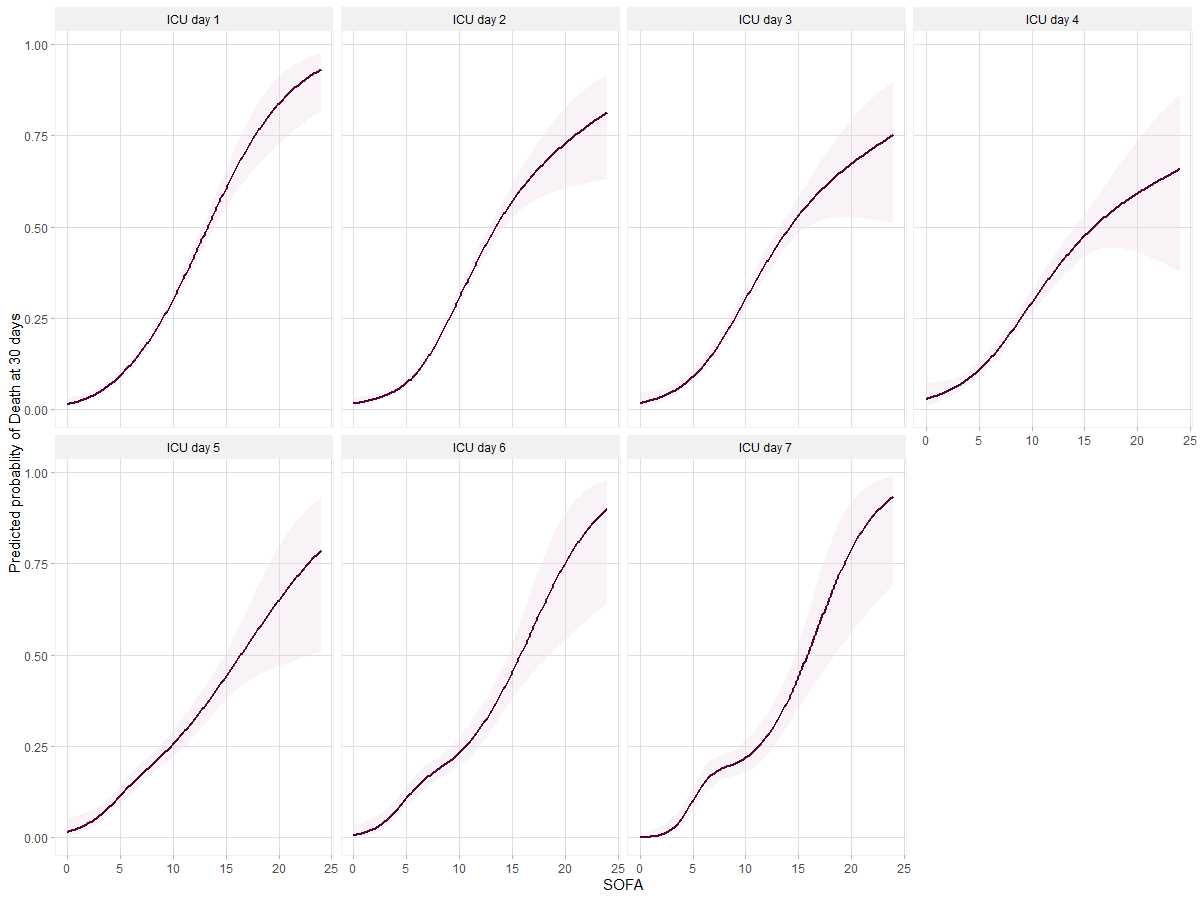


Figure S2. Predicted probability of death at 30 days per SOFA score on ICU-day 1 through 7. On day 1 the curve is S-shaped with increased mortality especially steep in the SOFA range about 7 to 15. For all curves the confidence interval is increased for higher SOFA scores mainly due to fewer observations.

Calibration of SOFA on ICU day 2 is shown in Figure S3, with predicted probabilities tracking the observed 30-day mortality closely. Discrimination was stable across the interval day 1 to 7, while estimates after day 15 were less precise due to smaller samples. As shown in Figure S4, the largest absolute increase in mortality per 1-point increase in SOFA occurs around scores 7 to 11. Interpretation of the extreme upper tails of the curves should be undertaken with caution due to sparse data in this range.


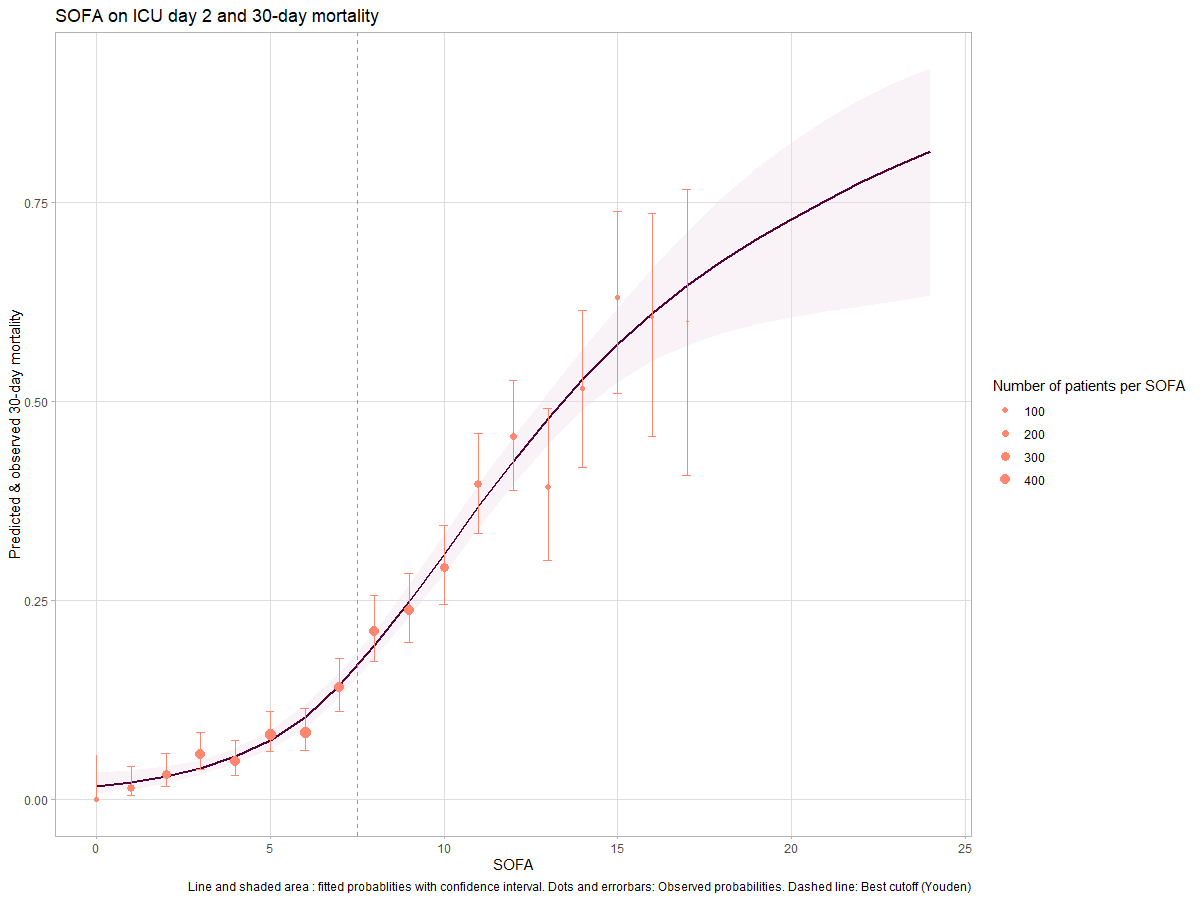


Figure S3: Predicted probabilities from SOFA on ICU day 2 and observed 30-day mortality. Line and shaded area: Fitted probabilities. Dots and errorbars: Observed probabilities, 95% CI. Dashed line: Best cutoff (Youden's criteria)


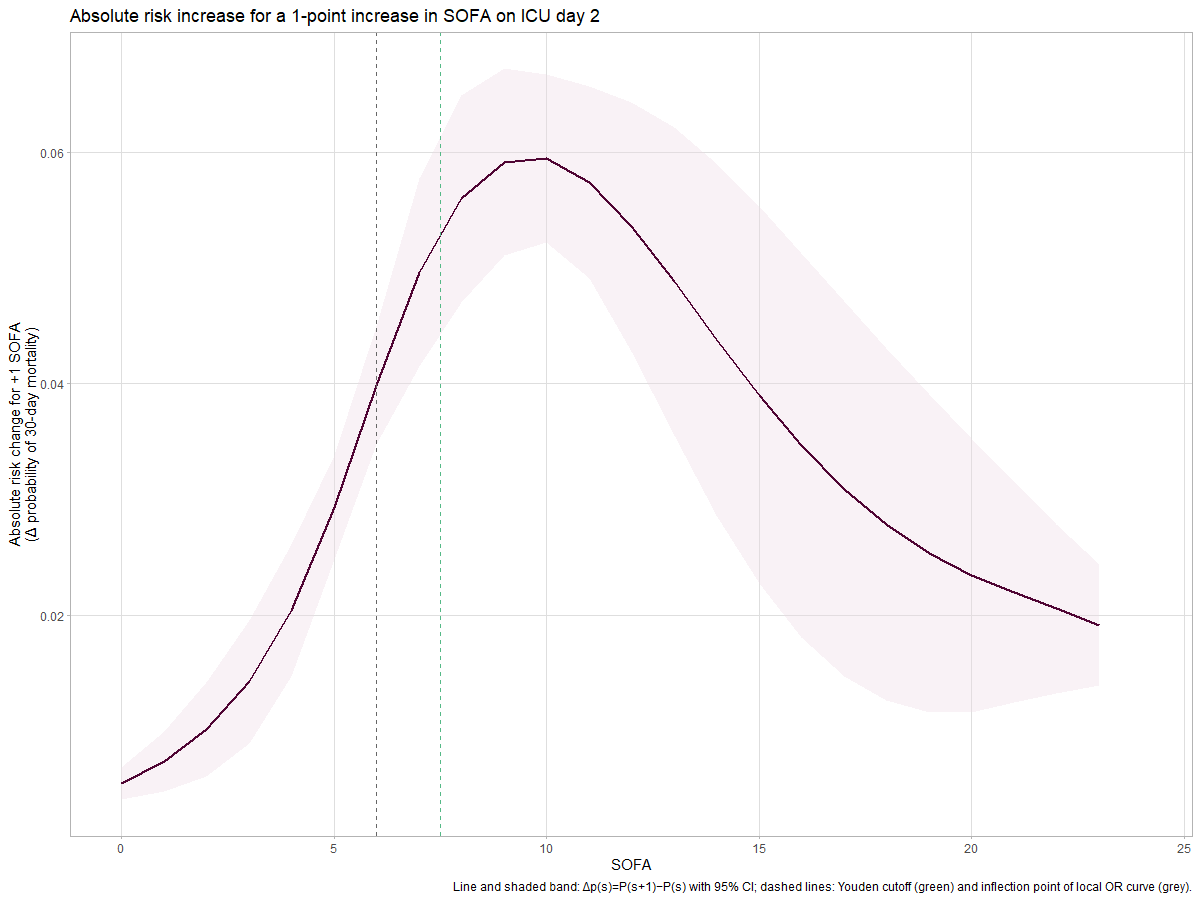


Figure S4: Estimated absolute risk increase of 30-day mortality for a 1-point increase in SOFA score on ICU day 2, as a function of SOFA score. Dashed lines: Youden cutoff (green) and inflection point from local Odds Ratio curve (grey).

^1^ NIELSEN, A. B. et al. Survival prediction in intensive-care units based on aggregation of long-term disease history and acute physiology: a retrospective study of the Danish National Patient Registry and electronic patient records. **Lancet Digit Health,** v. 1, n. 2, p. e78-e89, 06 2019. ISSN 2589-7500. Disponível em: < <https://www.ncbi.nlm.nih.gov/pubmed/33323232> >.

^2^ PANDHARIPANDE, P. P. et al. Derivation and validation of Spo2/Fio2 ratio to impute for Pao2/Fio2 ratio in the respiratory component of the Sequential Organ Failure Assessment score. **Crit Care Med,** v. 37, n. 4, p. 1317-21, Apr 2009. ISSN 1530-0293. Disponível em: < <https://www.ncbi.nlm.nih.gov/pubmed/19242333> >.

^3^ GORADIA, S. et al. Vasopressor dose equivalence: A scoping review and suggested formula. **J Crit Care,** v. 61, p. 233-240, Feb 2021. ISSN 1557-8615 (Electronic)

0883-9441 (Linking).

^4^ LAMBDEN, S. et al. The SOFA score-development, utility and challenges of accurate assessment in clinical trials. **Crit Care,** v. 23, n. 1, p. 374, Nov 27 2019. ISSN 1364-8535 (Print)

1364-8535.

^5^ VINCENT, J. L. et al. The SOFA (Sepsis-related Organ Failure Assessment) score to describe organ dysfunction/failure. On behalf of the Working Group on Sepsis-Related Problems of the European Society of Intensive Care Medicine. **Intensive Care Med,** v. 22, n. 7, p. 707-10, Jul 1996. ISSN 0342-4642. Disponível em: < <https://www.ncbi.nlm.nih.gov/pubmed/8844239> >.

^6^ HELLEBERG, J. et al. Beyond labels: determining the true type of blood gas samples in ICU patients through supervised machine learning. **BMC Med Inform Decis Mak,** v. 25, n. 1, p. 275, Jul 24 2025. ISSN 1472-6947. Disponível em: < <https://www.ncbi.nlm.nih.gov/pubmed/40707901> >.

^7^ VINCENT, J. L. et al. International study of the prevalence and outcomes of infection in intensive care units. **JAMA,** v. 302, n. 21, p. 2323-9, Dec 02 2009. ISSN 1538-3598. Disponível em: < <https://www.ncbi.nlm.nih.gov/pubmed/19952319> >.

^8^ BROWN, S. M. et al. Nonlinear Imputation of Pao2/Fio2 From Spo2/Fio2 Among Patients With Acute Respiratory Distress Syndrome. **Chest,** v. 150, n. 2, p. 307-13, Aug 2016. ISSN 1931-3543. Disponível em: < <https://www.ncbi.nlm.nih.gov/pubmed/26836924> >.

^9^ ZHAO, Y.; HRYNIEWICKI, M. K. **XGBOD: Improving Supervised Outlier Detection with Unsupervised Representation Learning**: IEEE 2018.

^10^ VINCENT, J. L. et al. Use of the SOFA score to assess the incidence of organ dysfunction/failure in intensive care units: results of a multicenter, prospective study. Working group on "sepsis-related problems" of the European Society of Intensive Care Medicine. **Crit Care Med,** v. 26, n. 11, p. 1793-800, Nov 1998. ISSN 0090-3493 (Print)

0090-3493 (Linking).

^12^ BREUNIG, M. M. et al. **LOF: Identifying Density-Based Local Outliers.** 2000.

^13^ TANG, J. et al. **Enhancing Effectiveness of Outlier Detections for Low Density Patterns**. Proceedings of the Sixth Pacific-Asia Conference on  Knowledge Discovery and Data Mining (PAKDD), Taipei, May. 2002.

^14^ KRIEGEL, H.-P. et al. **LoOP: local outlier probabilities**. Proceedings of the 18th ACM conference on Information and knowledge management. Hong Kong, China: Association for Computing Machinery**:** 1649–1652 p. 2009.

^15^ SOUIDEN, I.; BRAHMI, Z.; TOUMI, H. **A survey on outlier detection in the context of stream mining: review of existing approaches and recommadations**. International conference on intelligent systems design and applications: Springer, 2016. 372-383 p.

^16^ ALGHUSHAIRY, O. et al. A review of local outlier factor algorithms for outlier detection in big data streams. **Big Data and Cognitive Computing,** v. 5, n. 1, p. 1, 2020. ISSN 2504-2289.

^17^ HARRELL, F. E.; LEE, K. L.; MARK, D. B. Multivariable prognostic models: issues in developing models, evaluating assumptions and adequacy, and measuring and reducing errors. **Stat Med,** v. 15, n. 4, p. 361-87, Feb 28 1996. ISSN 0277-6715. Disponível em: < <https://www.ncbi.nlm.nih.gov/pubmed/8668867> >.
